# A comparison of five methods of measuring mammographic density: a case-control study

**DOI:** 10.1186/s13058-018-0932-z

**Published:** 2018-02-05

**Authors:** Susan M. Astley, Elaine F. Harkness, Jamie C. Sergeant, Jane Warwick, Paula Stavrinos, Ruth Warren, Mary Wilson, Ursula Beetles, Soujanya Gadde, Yit Lim, Anil Jain, Sara Bundred, Nicola Barr, Valerie Reece, Adam R. Brentnall, Jack Cuzick, Tony Howell, D. Gareth Evans

**Affiliations:** 1 Division of Informatics, Imaging and Data Sciences, Faculty of Biology, Medicine and Health, University of Manchester, Manchester Academic Health Science Centre, Stopford Building, Oxford Road, Manchester, M13 9PT UK; 2 Prevent Breast Cancer and Nightingale Breast Screening Centre, Manchester University NHS Foundation Trust, Manchester Academic Health Science Centre, Southmoor Road, Wythenshawe, Manchester, M23 9LT UK; 3 Arthritis Research UK Centre for Epidemiology, Centre for Musculoskeletal Research, Faculty of Biology, Medicine and Health, Manchester Academic Health Science Centre, University of Manchester, Oxford Road, Manchester, M13 9PT UK; 4 NIHR Manchester Musculoskeletal Biomedical Research Unit, Manchester University NHS Foundation Trust, Manchester Academic Health Science Centre, Manchester, M13 9WL UK; 5 Warwick Clinical Trials Unit, Division of Health Sciences, Warwick Medical School, University of Warwick, Coventry, CV4 7AL UK; 6 Department of Radiology, University of Cambridge, Addenbrooke’s Hospital, Cambridge, UK; 7 School of Medical Sciences, University of Manchester, Oxford Road, Manchester, UK; 8 Centre for Cancer Prevention, Wolfson Institute of Preventive Medicine, Queen Mary University of London, London, EC1M 6BQ UK; 9 The Christie NHS Foundation Trust, Manchester Academic Health Science Centre, Withington, Manchester, M20 4BX UK; 10 Genomic Medicine, Division of Evolution and Genomic Science, Manchester Academic Health Sciences Centre, University of Manchester and Manchester University NHS Foundation Trust, Manchester, M13 9WL UK

**Keywords:** Breast density, Case-control, Risk, Cancer, PROCAS

## Abstract

**Background:**

High mammographic density is associated with both risk of cancers being missed at mammography, and increased risk of developing breast cancer. Stratification of breast cancer prevention and screening requires mammographic density measures predictive of cancer. This study compares five mammographic density measures to determine the association with subsequent diagnosis of breast cancer and the presence of breast cancer at screening.

**Methods:**

Women participating in the “Predicting Risk Of Cancer At Screening” (PROCAS) study, a study of cancer risk, completed questionnaires to provide personal information to enable computation of the Tyrer-Cuzick risk score. Mammographic density was assessed by visual analogue scale (VAS), thresholding (Cumulus) and fully-automated methods (Densitas, Quantra, Volpara) in contralateral breasts of 366 women with unilateral breast cancer (cases) detected at screening on entry to the study (Cumulus 311/366) and in 338 women with cancer detected subsequently. Three controls per case were matched using age, body mass index category, hormone replacement therapy use and menopausal status. Odds ratios (OR) between the highest and lowest quintile, based on the density distribution in controls, for each density measure were estimated by conditional logistic regression, adjusting for classic risk factors.

**Results:**

The strongest predictor of screen-detected cancer at study entry was VAS, OR 4.37 (95% CI 2.72–7.03) in the highest vs lowest quintile of percent density after adjustment for classical risk factors. Volpara, Densitas and Cumulus gave ORs for the highest vs lowest quintile of 2.42 (95% CI 1.56–3.78), 2.17 (95% CI 1.41–3.33) and 2.12 (95% CI 1.30–3.45), respectively. Quantra was not significantly associated with breast cancer (OR 1.02, 95% CI 0.67–1.54). Similar results were found for subsequent cancers, with ORs of 4.48 (95% CI 2.79–7.18), 2.87 (95% CI 1.77–4.64) and 2.34 (95% CI 1.50–3.68) in highest vs lowest quintiles of VAS, Volpara and Densitas, respectively. Quantra gave an OR in the highest vs lowest quintile of 1.32 (95% CI 0.85–2.05).

**Conclusions:**

Visual density assessment demonstrated a strong relationship with cancer, despite known inter-observer variability; however, it is impractical for population-based screening. Percentage density measured by Volpara and Densitas also had a strong association with breast cancer risk, amongst the automated measures evaluated, providing practical automated methods for risk stratification.

**Electronic supplementary material:**

The online version of this article (10.1186/s13058-018-0932-z) contains supplementary material, which is available to authorized users.

## Background

High mammographic density, the relative proportion of fibroglandular to fatty tissue in the breast, reduces the effectiveness of mammographic screening [1–4] and increases risk of developing breast cancer [5, 6]. The relationship of density with risk was established using expert visual assessment of film mammograms [7], with computer-assisted methods providing more reproducible estimates [8, 9]. With increasing uptake of full-field digital mammography (FFDM), the association between automated density assessment methods and cancer risk is under investigation [10–12].

The most widely used method of assessing mammographic density in the USA is the Breast Imaging Reporting And Data System (BI-RADS) categorisation, where experts assign mammograms to one of four classes, the upper two being considered “dense”’ [13]. The class descriptors were changed in 2013 to better identify women whose cancers may be masked by dense parenchymal tissue [14]. Visual assessment of percentage density may be recorded on visual analogue scales (VAS), providing a continuous measure. This yielded a strong relationship with breast cancer risk for film mammograms, with an odds ratio (OR) of approximately 7 for 76–100% density relative to 0–25% [15]. Likewise, Boyd’s 6-class categorisation of percent visual density gave a relative risk in the highest category (> 75% dense) compared with the lowest of 6.05 (95% confidence interval (CI) = 2.82–12.97) in a case-control study with 354 cases [8]. Cumulus, a semi-automated thresholding method, was developed to improve reproducibility [8, 9] and has a well-established relationship with cancer risk [8, 12]. However, this method also requires trained observers, and whilst separating the breast from the mammogram background is reproducible, judgement of the best threshold to separate dense tissue from fat is less so. Boyd category, VAS and Cumulus are all relative, area-based methods, so density estimates can vary depending on breast positioning and patient weight [8, 16]. Weight change disproportionately alters the fatty component of the breast [17] and percentage density measures should be adjusted to take body mass index (BMI) into account [18].

Now that digital mammography is standard throughout the UK, volumetric measures of mammographic density, made by calibrating pixel values in the raw (“for processing”) FFDM image using a model of x-ray physics and imaging parameters [19], are now available. These can be expressed either in percentage terms (volumetric percent dense) or as absolute measures of dense and non-dense tissue.

The availability of fully automated density assessment paves the way for risk stratification in screening [20], allowing selection of the most appropriate imaging modality and screening frequency for the individual [21, 22]. The addition of mammographic density to breast cancer risk models based on other risk factors has demonstrated increased predictive power, depending on the method used for density estimation [23–25]. It is therefore important to determine which density methods are suitable for risk-adapted screening; more accurate risk prediction will enable better targeting of risk-reducing interventions including chemoprevention and lifestyle modification [26, 27].

A previous case-control analysis, carried out in London, compared density measured in the unaffected (contralateral) breast in 414 women diagnosed with unilateral breast cancer at one hospital with that of 685 unmatched controls attending routine breast screening. Comparing the highest percentage density quintile with the lowest, and adjusting for age, BMI and reproductive variables, the strongest association with risk of developing breast cancer was for Volpara, with an OR of 8.26 (95% CI 4.28–15.96), followed by Quantra, OR 3.94 (2.26–6.86) and Cumulus, OR 3.38 (2.00–5.72) [13]. However, mammographic density was assessed at the time of detection of cancer, so the ability of density to predict women who would later develop the disease was not assessed. Here we address this by evaluating the association between five mammographic density methods and the presence of cancer at the time of screening, and the association between four mammographic density methods and cancer detected subsequently, either between screening rounds or at a later screen, using data from the Predicting Risk of Cancer At Screening study (PROCAS) [20].

## Methods

### Study design

Women invited to the Greater Manchester Breast Screening Service for routine 3-yearly mammographic screening between October 2009 and March 2015 were also invited to participate in the “Predicting Risk Of Cancer At Screening” (PROCAS) study, which aimed to provide women with a personalised risk estimate of their breast cancer risk based on mammographic density and classic breast cancer risk factors obtained via a questionnaire and quantified by the Tyrer-Cuzick risk score [28]. After October 2012 only women attending their first (prevalent round) screen were invited. At the time of recruitment informed consent was obtained from all participants.

In order to assess density using fully automated methods, the raw FFDM (for processing) image data from GE Senographe Essential mammography systems was obtained. Cancers (invasive and ductal carcinoma in situ) were identified through hospital records or through the North West Cancer Intelligence Service; women who moved out the area were considered ineligible. Two case-control datasets were created. In study 1, cases were women with breast cancer detected at the screen on entry to PROCAS and in study 2 cases were women who were breast cancer free at the screen on entry to PROCAS but had breast cancer detected subsequently, either between screening rounds or at a later screen. In these women we analysed the density of the screen on entry to PROCAS.

Three controls without cancer were matched to each cancer case based on age (±12 months), BMI category (missing, < 24.9, 25.0–29.9, 30+ kg/m^2^), hormone replacement therapy (HRT) use (current vs never/ever) and menopausal status (premenopausal, perimenopausal or postmenopausal). In both studies all controls had a subsequent cancer-free screening mammogram so it was unlikely that early signs of cancer were visible, and in study 2, controls were also matched on year of mammogram at entry.

### Mammographic density measurement

#### Visual estimation of percentage density

Processed FFDM images were displayed on Planar Dome E5 5MP self-calibrating high-resolution monitors. Two of nineteen readers (usually a consultant radiologist or breast physician and an advanced practitioner radiographer) independently recorded density estimates on a paper form showing four 10-cm horizontal VAS, one for each view, labelled 0% and 100% at the ends of the scale. Forms were read using custom software and visual percentage density calculated. VAS readings were averaged between readers and views, and analysed in quintiles and as Boyd categories (0%, > 0–10%, > 10–25%, > 25–50%, > 50–75% and > 75%) [8]. Due to the small number of cases in the highest category (three in study 1 and six in study 2), the top two Boyd categories were combined for analysis. Intra-observer and inter-observer agreement for 120 mammograms randomly selected across deciles of VAS density scores, from the PROCAS study, were assessed by 11 readers, on two occasions, 3 years apart. The majority of readers had excellent intra-observer agreement (intraclass correlation coefficient (ICC) > 0.80), and inter-observer agreement for consistency was excellent (ICC = 0.82) and was substantial for absolute agreement (ICC = 0.69) [29].

#### Cumulus

Cumulus (Sunnybrook Health Sciences Centre, Toronto, ON, Canada) density assessment was undertaken by a single reader (JS) trained in August 2010 and validated by a member of the PROCAS team (JW) who had herself been trained by the group that developed the software. Reader performance was validated on test sets of data developed for this purpose by the trainers. Processed FFDM images were analysed. Cumulus was undertaken on a single contralateral mediolateral oblique (MLO) view of a subset of the study 1 dataset comprising 311 screen-detected cancers and their matched controls. The reader was blind to case-control status.

#### Quantra™

Quantra version 2.0 (Hologic Inc, Bedford, MA, USA) was used to assess density from the raw FFDM images for each view, each breast and each woman, giving breast and fibroglandular tissue volume (cm^3^), and the dense tissue area as a percentage of breast volume. It also provides a quantized BI-RADS-like score for each view and per breast.

#### Volpara™

Volpara Density Algorithm 1.5.0 (Volpara Health Technologies, Wellington, New Zealand) was also used to assess density from the raw FFDM images for each view, giving breast volume and fibroglandular tissue volume (cm^3^) and percentage density by volume. Volpara provided a macro, which produced per-patient results including Volpara Density Grade (VDG 4^th^ and 5^th^ Edition), designed to correlate with BI-RADS 4^th^ and 5^th^ Edition [15]. This also computes the percentage density of the two breasts following outlier removal.

#### Densitas™

Densitas version 2.0.0 (Densitas Inc, Halifax, NS, Canada) analyses processed FFDM images, giving breast and fibroglandular area (cm^2^) and percentage density by area for each image and per patient. It also produces per-patient measures of BIRADS 4^th^ and 5^th^ edition [15].

### Statistical methods

In study 1, mammographic density was assessed in the contralateral breast in women with cancer and the breast on the same side in matched controls, whereas in study 2, density was assessed in both breasts at entry to PROCAS and the average was used.

Categorical data were compared using the chi-square test for proportions. For ordinal variables, a chi-square test for trend was also conducted. Continuous variables were assessed by the median and Mann-Whitney U test.

The relationship between density assessment and case-control status was analysed using conditional logistic regression. Density measures were modelled as quintiles based on the density distributions of controls, and also as continuous measures, transformed to approximately follow a normal distribution (square root transformation for VAS and Cumulus, and a logarithm transformation for Volpara, Quantra and Densitas). Univariate models were fitted initially, and multivariate models fitted to adjust for the logarithm 10-year Tyrer-Cuzick (v.6) risk score. In study 2 we also adjusted for parity, due to imbalance between cases and controls. We also performed an analysis in a subset of women who had been assessed using all density methods to determine which model performed best and differences between models were compared using the likelihood-ratio chi square. The matched concordance (mC) index, a modification of the concordance index (or area under the receiving operator characteristic curve (AUC)) for matched case-control studies, gives an average concordance index within matched groups (where 1.0 would indicate perfect discrimination after allowing for matching factors) with empirical bootstrap confidence intervals [30], was calculated to compare the discrimination performance of risk factors. All *p* values were two-sided. Analysis was performed in SPSS version 22 [31] and R 3.3.1 [32].

## Results

Of the 57,905 women recruited to PROCAS, raw FFDM image data were available for 44,658 women (77%). Unavailability of raw FFDM images was predominantly due to the use of film mammography initially. There were 1004 cases of cancer occurring after consent up to November 2015, of which 704 were included in the analysis. The excluded women comprised 39 women with a pre-existing diagnosis of breast cancer, 13 with synchronous bilateral breast cancer, 118 with film mammograms and 130 with FFDM but for which raw image data was unavailable. Of the 704 women eligible for the analysis, 366 were women with breast cancer detected at the screen on entry to PROCAS (study 1) and 338 were women who were found to be breast cancer free at the screen on entry to PROCAS but had breast cancer detected subsequently, either between screening rounds or at a later screen (study 2). Of the latter, 114 women developed an interval cancer within 5–46 months of entry (IQR 13–31) and 224 women had breast cancer detected at a subsequent screen 17–55 months after entry (IQR 35–38).

Matching was satisfactory for both studies (Table 1). There was a difference in 10-year Tyrer-Cuzick score, with the score higher in cases (study 1, 2.95 vs 2.72, *p* = 0.003; study 2: 2.91 vs 2.63, *p* < 0.001). The reported rate of a previous breast biopsy in cases was 17.8% (study 1) and 22.5% (study 2), and in controls it was 14.5% (study 1) and 15.1% (study 2). The difference in biopsy rate between cases and controls was statistically significant in study 2 (*p* = 0.005), but was similar (in study 2) to the PROCAS study as a whole. In study 1 significantly fewer cases than controls reported being of “white” ethnic origin (91.3% vs 94.5%, *p* = 0.003), and fewer cases than controls reported having children in study 2 (85.8% vs 90.2%, *p* = 0.023).

**Table 1:** Demographics of study participants at time of recruitment to the Predicting Risk of Cancer At Screening (PROCAS) study*

	Study 1					Study 2					PROCAS	
	Control subjects		Case subjects			Control subjects		Case subjects			(*n* = 57902)	
	(*n* = 1098)		(*n* = 366)			(*n* = 1014)		(*n* = 338)				
	Number	Percent	Number	Percent	*P* value	Number	Percent	Number	Percent	*P* value	Number	Percent
Age at consent (years)												
< 50	117	10.7	40	10.9		50	4.9	17	5.0		7173	12.4
50–54	316	28.8	104	28.4		203	20.0	66	19.5		16962	29.3
55–59	157	14.3	53	14.5		179	17.7	61	18.0		11046	19.1
60–64	237	21.6	79	21.6		315	31.1	105	31.1		11223	19.4
65–69	197	17.9	66	18.0		210	20.7	70	20.7		8552	14.8
70+	74	6.7	24	6.6	1.000	57	5.6	19	5.6	1.000	2946	5.1
Median (IQR)	58	51–64	58	51–64	0.988	61	55–65	61	55–65	0.965	57	51–63
Menopausal status												
Perimenopausal	170	15.5	57	15.6		145	14.3	49	14.5		10760	18.6
Postmenopausal	747	68.0	248	67.8		769	75.8	256	75.7		37201	64.2
Premenopausal	136	12.4	46	12.6		67	6.6	22	6.5		6869	11.9
Unknown	45	4.1	15	4.1	1.000	33	3.3	11	3.3	1.000	3072	5.3
HRT use												
Never	699	63.7	250	68.3		518	51.1	177	52.4		36505	63.0
Previous	342	31.1	93	25.4		379	37.4	122	36.1		16438	28.4
Current	56	5.1	19	5.2		110	10.8	37	10.9		4421	7.6
Unknown	1	0.1	4	1.1	0.302^a^	7	0.7	2	0.6	0.972	538	0.9
BMI (kg/m^2^)												
< 25	345	31.4	112	30.6		334	32.9	108	32.0		20774	35.8
25–29	353	32.1	122	33.3		346	34.1	118	34.9		18969	32.8
≥ 30	327	29.8	105	28.7		272	26.8	88	26.0		14256	24.6
Unknown	73	6.6	27	7.4	0.918	62	6.1	24	7.1	0.904	3933	6.8
Median (IQR)	27.2	24.0–31.2	27.8	24.0–30.8	0.581	26.6	23.9–30.5	26.6	23.9–30.5	0.517	26.4	23.6–30.3
Ethnic origin												
White	1038	94.5	334	91.3		924	91.1	304	89.9		52689	91.0
Other/unknown	50	4.6	32	8.7	0.003	90	8.9	34	10.1	0.511	5213	9.0
Year of mammogram												
2009	22	2.0	0	0.0							2372	4.1
2010	607	55.3	43	11.7		196	19.3	70	20.7		14761	25.5
2011	396	36.1	130	35.5		553	54.5	177	52.4		18350	31.7
2012	70	6.4	129	35.2		264	26.0	86	25.4		14214	24.5
2013	3	0.3	30	8.2		1	0.1	3	0.9		3499	6.0
2014	0	0.0	33	9.0		0	0.0	2	0.6		3804	6.6
2015	0	0.0	1	0.3	< 0.001^b^					0.769^c^	900	1.6
Initial Tyrer-Cuzick (10-year %)												
Median (IQR)	2.72	2.18 – 3.52	2.95	2.29–3.90	0.003	2.63	2.10–3.47	2.91	2.25–4.02	< 0.001	2.67	2.12–3.47
Family history												
None	803	73.1	249	68.0		754	74.4	229	67.8		42367	73.2
FDR only	104	9.5	42	11.5		107	10.6	47	13.0		5749	9.9
SDR only	147	13.4	51	13.9		126	12.4	42	12.4		7998	13.8
FDR and SDR	44	4.0	24	6.6	0.112	27	2.7	20	5.9	0.008	1788	3.1
Current alcohol use												
No	283	25.8	89	24.3		273	26.9	93	27.5		15815	27.3
Yes	797	72.6	269	73.5		726	71.6	241	71.3		41130	71.0
Unknown	18	1.6	8	2.2	0.697	15	1.5	4	1.2	0.908	957	1.7
Any children												
No	128	11.7	43	11.7		98	9.7	45	13.3		7384	12.7
Yes	970	88.3	323	88.3		915	90.2	290	85.8		50411	87.1
Unknown					0.963	1	0.1	3	0.9	0.023^d^	107	0.2
Prior biopsy of breast												
No	910	82.9	284	77.6		837	82.5	252	74.6		47359	81.8
Yes	159	14.5	65	17.8		153	15.1	76	22.5		8911	15.4
Unknown	29	2.6	17	4.6	0.041	24	2.4	10	3.0	0.005	1632	2.8

^*^In study 1 cases are women with breast cancer detected at first screen on entry to the PROCAS study and in study 2 cases are women with breast cancer detected within PROCAS at a subsequent screen or between screening rounds

*IQR* interquartile range, *HRT* hormone replacement therapy, *BMI* body mass index, *FDR* first-degree relative, *SDR* second-degree relative

^a^Excludes unknown

^b^2013-2015 combined

^c^2012-2014 combined

^d^Unknown combined with no children

In study 1, VAS results were missing for 46 cases of cancer, Quantra failed to produce results for one case and one control, Volpara failed for one case, and Densitas failed for 6 cases and 62 controls. In study 2 there were missing density results for two cases of cancer assessed by VAS, for one case and one control assessed by Quantra and for 7 cases and 34 controls assessed by Densitas.

### Study 1: screen-detected cancers

**Fig. 1:**
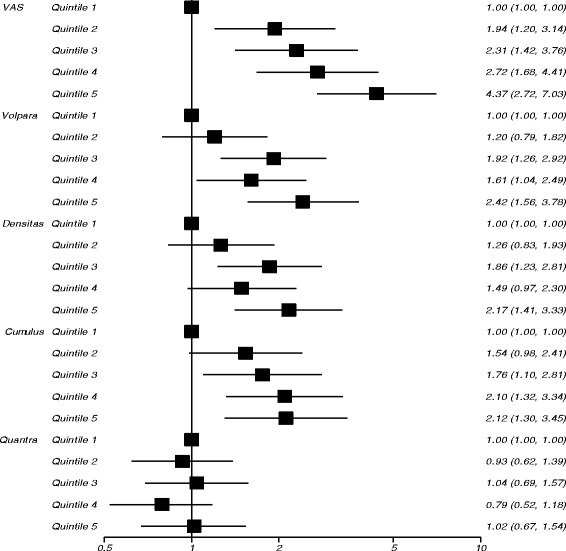
Risk of developing cancer (odds ratios on a logarithm scale) by quintiles of percent density measures in study 1

In study 1 after full adjustment, the strongest predictor of breast cancer risk was visually assessed density (Table 2, Fig. 1), with an odds ratio (OR) of 4.37 (95% CI 2.72–7.03) in the highest quintile of density compared with the lowest. When quantized in Boyd categories (Table 3), the adjusted OR of those with greater than 50% density was 6.73 (95% CI 3.64–12.45) compared to those with density 10% or lower. Volpara percent density provided the next strongest association with cancer, with an OR for the highest quintile of 2.42 (95% CI 1.56–3.78) (Table 2, Fig. 1). When quantized in Volpara Density Grades (VDG 5^th^ edition), the OR of VDG4 was 4.39 (95% CI 2.28–8.48) compared with VDG1 (Table 3). Both visually assessed density and Volpara percent density showed a significant and clear trend with increasing density (χ2 trend 35.6, *p* < 0.001 and 11.2, *p* < 0.001, respectively). Percent density measured by Densitas and Cumulus was also statistically significant (Table 2, Fig. 1), with ORs of 2.17 (95% CI 1.41–3.33) and 2.12 (95% CI 1.30–3.45), respectively in the highest quintile of percent density compared with the lowest, and for Quantra there was no significant association (OR = 1.02, 95% CI 0.67–1.54). The relationship with dense volume is shown in Table 2; generally associations tended to be slightly lower than those for percent density. In the subset of women with all density measures VAS was a significantly better predictor of breast cancer risk than all other methods (Table 2, Additional file 1: Table S2). The matched concordance index for VAS was 0.651 (95% CI 0.611–0.691) demonstrating better discrimination between cases and controls than all other methods (Table 4).

**Table 2:** Risk of developing breast cancer by density measures (highest versus the lowest quintile^a^ (referent))

					Subset with data for all methods^b^			
	Univariate		Adjusted^c^		Univariate		Adjusted^c^	
	OR	95% CI	OR	95% CI	OR	95% CI	OR	95% CI
Study 1								
	1.00	(referent)	1.00	(referent)	1.00	(referent)	1.00	(referent)
VAS (%)	*4.45*	*(2.77–7.15)*	*4.37*	*(2.72–7.03)*	*5.61*	*(3.29–9.56)*	*5.44*	*(3.18–9.29)*
Volpara gland volume (cm^3^)	*2.13*	*(1.40–3.24)*	*2.09*	*(1.37–3.18)*	*2.00*	*(1.24–3.21)*	*1.97*	*(1.22–3.18)*
Volpara breast density (%)	*2.44*	*(1.57–3.80)*	*2.42*	*(1.56–3.78)*	*2.38*	*(1.45–3.91)*	*2.41*	*(1.46–3.97)*
Cumulus dense area (cm^2^)	*2.11*	*(1.32–3.38)*	*2.15*	*(1.34–3.45)*	*2.08*	*(1.28–3.27)*	*2.12*	*(1.30–3.45)*
Cumulus breast density (%)	*2.09*	*(1.29–3.40)*	*2.12*	*(1.30–3.45)*	*2.20*	*(1.32–3.64)*	*2.23*	*(1.34–3.71)*
Quantra gland volume (cm^3^)	0.86	(0.58–1.30)	0.83	(0.55–1.25)	0.72	(0.45–1.15)	0.71	(0.44–1.13)
Quantra breast density (%)	1.05	(0.70–1.59)	1.02	(0.67–1.54)	1.11	(0.70–1.77)	1.08	(0.68–1.72)
Densitas dense area (cm^2^)	1.44	(0.96–2.16)	1.41	(0.93–2.12)	*1.62*	*(1.01–2.59)*	*1.61*	*(1.00–2.58)*
Densitas breast density (%)	*2.30*	*(1.50–3.52)*	*2.17*	*(1.41–3.33)*	*2.19*	*(1.35–3.56)*	*2.10*	*(1.29–3.41)*
Study 2								
	1.00	(referent)	1.00	(referent)	1.00	(referent)	1.00	(referent)
VAS (%)	*4.54*	*(2.86–7.22)*	*4.48*	*(2.79–7.18)*	*4.41*	*(2.76–7.06)*	*4.36*	*(2.70–7.04)*
Volpara gland volume (cm^3^)	*2.72*	*(1.79–4.14)*	*2.66*	*(1.74–4.08)*	*2.71*	*(1.77–4.14)*	*2.65*	*(1.72–4.09)*
Volpara breast density (%)	*2.78*	*(1.74–4.44)*	*2.87*	*(1.77–4.64)*	*2.61*	*(1.62–4.19)*	*2.71*	*(1.67–4.39)*
Quantra gland volume (cm^3^)	1.36	(0.90–2.06)	1.28	(0.84–1.95)	1.32	(0.87–2.01)	1.24	(0.80–1.90)
Quantra breast density (%)	1.32	(0.86–2.03)	1.32	(0.85–2.05)	1.30	(0.84–2.00)	1.32	(0.85–2.05)
Densitas dense area (cm^2^)	*2.34*	*(1.56–3.52)*	*2.23*	*(1.48–3.38)*	*2.29*	*(1.50–3.50)*	*2.14*	*(1.40–3.29)*
Densitas breast density (%)	*2.45*	*(1.57–3.82)*	*2.34*	*(1.50–3.68)*	*2.44*	*(1.56–3.80)*	*2.34*	*(1.49–3.66)*

*OR* odds ratio, *CI* confidence interval, *VAS* visual analogue scale, results in italics indicate statistically significant results (*p*<0.05)

^a^Quintiles based on distribution amongst controls

^b^Study 1: 239 cases with 3 controls, 62 with 2 controls and 2 with 1 control; study 2: 296 cases with 3 controls, 31 with 2 controls and 2 with 1 control

^c^Adjusted for Tyrer-Cuzick score; study 2 also adjusted for parity

Figures in italics denote statistically significant results *p*<0.01

**Table 3:** Density measures by categorical scales

									Subset with data for all methods^b^							
	Controls		Cases		Univariate		Adjusted^a^		Controls		Cases		Univariate		Adjusted^a^	
	*N*	Percent	*N*	Percent	OR	95% CI	OR	95% CI	*N*	Percent	*N*	Percent	OR	95% CI	OR	95% CI
Study 1																
VAS (mean) - Boyd categories (combining > 50–75% and > 75%)																
≥ 0–10%	208	(18.9)	29	(9.1)	1.00	(referent)	1.00	(referent)	176	(21.1)	25	(8.3)	1.00	(referent)	1.00	(referent)
> 10–25%	436	(39.7)	115	(35.9)	*2.17*	*(1.37–3.42)*	*2.16*	*(1.37–3.40)*	331	(39.7)	111	(36.9)	*2.67*	*(1.63–4.38)*	*2.65*	*(1.62–4.34)*
> 25–50%	379	(34.5)	133	(41.6)	*3.25*	*(2.05–5.18)*	*3.20*	*(2.01–5.09)*	277	(33.3)	125	(41.5)	*3.95*	*(2.38–6.54)*	*3.84*	*(2.31–6.36)*
> 50%	75	(6.8)	43	(13.4)	*6.92*	*(3.74–12.79)*	*6.73*	*(3.64–12.45)*	49	(5.9)	40	(13.3)	*8.30*	*(4.28–16.07)*	*8.02*	*(4.13–15.56)*
Volpara Density Grades 4th Edition																
1 (<4.5)	496	(45.2)	124	(34.0)	1.00	(referent)	1.00	(referent)	400	(48.0)	108	(35.9)	1.00	(referent)	1.00	(referent)
2 (4.5–<7.5)	339	(30.9)	131	(35.9)	*1.78*	*(1.31–2.41)*	*1.74*	*(1.29–2.37)*	258	(31.0)	113	(37.5)	*1.90*	*(1.35–2.66)*	*1.88*	*(1.34–2.63)*
3 (7.5–<15.5)	229	(20.9)	85	(23.3)	*1.85*	*(1.29–2.64)*	*1.86*	*(1.30–2.68)*	153	(18.4)	64	(21.3)	*1.92*	*(1.28–2.90)*	*1.94*	*(1.29–2.94)*
4 (15.5+)	34	(3.1)	25	(6.8)	*4.18*	*(2.24–7.77)*	*4.07*	*(2.18–7.60)*	22	(2.6)	16	(5.3)	*3.73*	*(1.80–7.75)*	*3.58*	*(1.72–7.44)*
Volpara Density Grades 5th Edition																
1 (< 3.5)	274	(25.0)	62	(17.0)	1.00	(referent)	1.00	(referent)	216	(25.9)	50	(16.6)	1.00	(referent)	1.00	(referent)
2 (3.5–<7.5)	561	(51.1)	193	(52.9)	*1.68*	*(1.20–2.34)*	*1.68*	*(1.20–2.35)*	442	(53.1)	171	(56.8)	*1.88*	*(1.29–2.73)*	*1.89*	*(1.30–2.75)*
3 (7.5–<15.5)	229	(20.9)	85	(23.3)	*2.00*	*(1.32–3.02)*	*2.04*	*(1.34–3.09)*	153	(18.4)	64	(21.3)	*2.22*	*(1.38–3.57)*	*2.27*	*(1.41–3.66)*
4 (15.5+)	34	(3.1)	25	(6.8)	*4.44*	*(2.31–8.54)*	*4.39*	*(2.28–8.48)*	22	(2.6)	16	(5.3)	*4.26*	*(1.98–9.17)*	*4.14*	*(1.92–8.95)*
Quantized density (BIRADS)																
1	54	(4.9)	27	(7.4)	1.00	(referent)	1.00	(referent)	47	(5.6)	21	(7.0)	1.00	(referent)	1.00	(referent)
2	682	(62.2)	220	(60.3)	0.63	(0.39–1.02)	*0.61*	*(0.38–0.99)*	530	(63.6)	193	(64.1)	0.85	(0.50–1.44)	0.80	(0.47–1.37)
3	317	(28.9)	102	(27.9)	0.63	(0.37–1.05)	*0.58*	*(0.34–0.99)*	226	(27.1)	75	(24.9)	0.77	(0.43–1.38)	0.71	(0.39–1.28)
4	44	(4.0)	16	(4.4)	0.71	(0.33–1.52)	0.62	(0.29–1.33)	30	(3.6)	12	(4.0)	0.92	(0.39–2.17)	0.79	(0.33–1.88)
Densitas: BIRADS 4																
0–24	387	(37.4)	105	(35.2)	1.00	(referent)	1.00	(referent)	326	(39.1)	94	(31.2)	1.00	(referent)	1.00	(referent)
25–50	599	(57.8)	219	(60.8)	*1.53*	*(1.15–2.04)*	*1.48*	*(1.11–1.98)*	470	(56.4)	186	(61.8)	*1.53*	*(1.12–2.08)*	*1.48*	*(1.09–2.03)*
51+	50	(4.8)	36	(10.0)	*3.46*	*(2.04–5.87)*	*3.21*	*(1.88–5.47)*	35	(4.2)	21	(7.0)	*2.47*	*(1.34–4.55)*	*2.33*	*(1.26–4.30)*
Densitas: BIRADS 5																
A	194	(18.7)	46	(12.8)	1.00	(referent)	1.00	(referent)	171	(20.5)	37	(12.3)	1.00	(referent)	1.00	(referent)
B	552	(53.3)	191	(53.1)	*1.58*	*(1.08–2.30)*	*1.50*	*(1.03–2.19)*	446	(53.5)	174	(57.8)	*1.97*	*(1.29–2.99)*	*1.87*	*(1.23–2.85)*
C	278	(26.8)	117	(32.5)	*2.01*	*(1.33–3.04)*	*1.92*	*(1.27–2.91)*	206	(24.7)	85	(28.2)	*2.12*	*(1.34–3.37)*	*2.03*	*(1.28–3.23)*
D	12	(1.2)	6	(1.7)	2.79	(0.94–8.24)	2.50	(0.84–7.48)	8	(1.0)	5	(1.7)	*3.71*	*(1.09–12.66)*	*3.30*	*(0.95–11.42)*
Study 2																
VAS (mean) - Boyd categories (combining >50–75% and >75%)																
≥ 0–10%	193	(19.0)	34	(10.1)	1.00	(referent)	1.00	(referent)	180	(18.7)	32	(9.7)	1.00	(referent)	1.00	(referent)
> 10–25%	401	(39.5)	103	(30.5)	*1.61*	*(1.05–2.49)*	*1.55*	*(1.00–2.40)*	383	(39.8)	100	(30.4)	*1.61*	*(1.03–2.50)*	1.55	(0.99–2.42)
> 25–50%	356	(35.1)	154	(45.6)	*2.92*	*(1.89–4.52)*	*2.87*	*(1.85–4.46)*	329	(34.2)	152	(46.2)	*3.02*	*(1.94–4.71)*	*2.97*	*(1.90–4.67)*
> 50%	64	(6.3)	45	(13.3)	*5.52*	*(3.08–9.91)*	*5.45*	*(3.00–9.89)*	60	(6.2)	45	(13.7)	*5.65*	*(3.12–10.23)*	*5.63*	*(3.07–10.3)*
Volpara Density Grades 4th Edition																
1 (< 4.5)	474	(46.7)	126	(37.3)	1.00	(referent)	1.00	(referent)	447	(46.5)	123	(37.4)	1.00	(referent)	1.00	(referent)
2 (4.5– < 7.5)	336	(33.1)	120	(35.5)	*1.45*	*(1.07–1.96)*	*1.44*	*(1.06–1.95)*	315	(32.7)	118	(35.9)	*1.44*	*(1.06–1.95)*	*1.43*	*(1.05–1.95)*
3 (7.5– < 15.5)	167	(16.5)	73	(21.6)	*1.96*	*(1.34–2.87)*	*2.08*	*(1.40–3.08)*	156	(16.2)	69	(21.0)	*1.81*	*(1.23–2.67)*	*1.92*	*(1.29–2.86)*
4 (15.5+)	37	(3.6)	19	(5.6)	*2.47*	*(1.30–4.70)*	*2.61*	*(1.34–5.10)*	34	(3.5)	19	(5.8)	*2.56*	*(1.33–4.91)*	*2.73*	*(1.38–5.38)*
Volpara Density Grades 5th Edition																
1 (<3.5)	205	(20.2)	47	(13.9)	1.00	(referent)	1.00	(referent)	195	(20.3)	47	(14.3)	1.00	(referent)	1.00	(referent)
2 (3.5–<7.5)	570	(56.2)	189	(55.9)	*1.55*	*(1.07–2.24)*	*1.53*	*(1.06–2.23)*	533	(55.4)	184	(55.9)	*1.51*	*(1.04–2.19)*	*1.51*	*(1.04–2.21)*
3 (7.5–<15.5)	193	(19.0)	78	(23.1)	*2.09*	*(1.33–3.28)*	*2.16*	*(1.36–3.43)*	181	(18.8)	74	(22.5)	*1.92*	*(1.22–3.04)*	*1.99*	*(1.25–3.17)*
4 (15.5+)	46	(4.5)	24	(7.1)	*2.92*	*(1.53–5.58)*	*3.00*	*(1.54–5.86)*	43	(4.5)	24	(7.3)	*2.82*	*(1.47–5.40)*	*2.91*	*(1.48–5.72)*
Quantized density (BIRADS)																
1	32	(3.2)	12	(3.6)	1.00	(referent)	1.00	(referent)	32	(3.3)	12	(3.6)	1.00	(referent)	1.00	(referent)
2	654	(64.5)	197	(58.5)	0.81	(0.39–1.70)	0.87	(0.42–1.82)	616	(64.0)	192	(58.4)	0.82	(0.39–1.71)	0.88	(0.42–1.83)
3	287	(28.3)	108	(32.0)	1.05	(0.49–2.27)	1.12	(0.52–2.41)	267	(27.8)	105	(31.9)	1.04	(0.48–2.23)	1.11	(0.52–2.39)
4	40	(3.9)	20	(5.9)	1.51	(0.59–3.88)	1.54	(0.59–4.01)	37	(3.8)	20	(6.1)	1.50	(0.58–3.87)	1.54	(0.59–4.02)
Densitas: BIRADS 4																
0–24	354	(36.1)	95	(28.7)	1.00	(referent)	1.00	(referent)	345	(35.9)	95	(28.9)	1.00	(referent)	1.00	(referent)
25–50	586	(59.8)	208	(62.8)	*1.37*	*(1.02–1.83)*	*1.34*	*(1.00–1.80)*	568	(59.0)	207	(62.9)	*1.37*	*(1.02–1.83)*	*1.34*	*(1.00–1.80)*
51+	40	(4.1)	28	(8.5)	*2.94*	*(1.65–5.24)*	*2.83*	*(1.55–5.16)*	39	(4.1)	27	(8.2)	*2.86*	*(1.60–5.11)*	*2.76*	*(1.51–5.05)*
Densitas: BIRADS 5																
A	96	(9.8)	25	(7.6)	1.00	(referent)	1.00	(referent)	94	(9.8)	25	(7.6)	1.00	(referent)	1.00	(referent)
B	574	(58.6)	185	(57.9)	1.25	(0.77–2.01)	1.20	(0.74–1.94)	558	(58.0)	185	(56.2)	1.25	(0.77–2.01)	1.20	(0.74–1.94)
C	294	(30.0)	110	(33.2)	1.47	(0.88–2.45)	1.46	(0.87–2.45)	284	(29.5)	108	(32.8)	1.46	(0.88–2.44)	1.45	(0.87–2.44)
D	16	(1.6)	11	(3.3)	*2.90*	*(1.14–7.42)*	2.59	(0.99–6.78)	16	(1.7)	11	(3.3)	*2.90*	*(1.13–7.41)*	2.58	(0.99–6.76)

*N* number, *OR* odds ratio, *CI* confidence interval, *VAS* visual analogue scale, *BIRADS* Breast Imaging Reporting and Data System, results in italics indicate statistically significant results (*p*<0.05)

^a^Adjusted for Tyrer-Cuzick score; study 2 also adjusted for parity

^b^Study 1: 239 cases with 3 controls, 62 with 2 controls and 2 with 1 control; study 2: 296 cases with 3 controls, 31 with 2 controls and 2 with 1 control

**Table 4:** Matched concordance index (mC)

	Study 1			Study 2		
	mC^a^	95% CI		mC^b^	95% CI	
VAS (%)	0.651	0.611	0.691	0.647	0.607	0.688
Volpara breast density (%)	0.571	0.528	0.618	0.575	0.534	0.615
Volpara gland volume (cm^3^)	0.553	0.513	0.591	0.586	0.546	0.627
Quantra breast density (%)	0.510	0.469	0.552	0.543	0.504	0.584
Quantra gland volume (cm^3^)	0.487	0.447	0.528	0.531	0.490	0.574
Densitas breast density (%)	0.571	0.526	0.612	0.587	0.548	0.628
Densitas dense area (cm^2^)	0.535	0.496	0.574	0.577	0.537	0.616
Cumulus breast density (%)	0.582	0.541	0.623	-	-	-
Cumulus dense area (cm^2^)	0.558	0.516	0.599	-	-	-

*CI* confidence interval, *VAS* visual analogue scale

^a^Study 1: 239 cases with 3 controls, 62 with 2 controls, 2 with 1 control

^b^Study 2: 296 with 3 controls, 31 with 2 controls, 2 with 1 control

### Study 2: prior mammograms

**Fig. 2:**
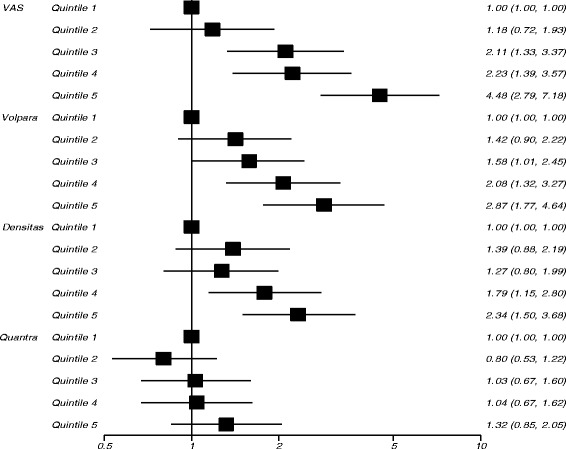
Risk of developing cancer (odds ratios on a logarithm scale) by quintiles of percent density measures for study 2

In study 2 visually assessed density had the strongest association with subsequent cancer in the fully adjusted models, with an OR of 4.48 (95% CI 2.79–7.18) in the highest quintile of density compared with the lowest (Table 2). When quantized in Boyd categories (Table 3), the OR of those with density > 50% was 5.45 (95% CI 3.00–9.89) compared to those with density ≤ 10%. Volpara percent density had the next strongest association with cancer, with an OR for the highest quintile of 2.87 (95% CI 1.77–4.64) (Table 2, Fig. 2). When quantized in Volpara Density Grades (5^th^ Edition), the OR of VDG4 was 3.00 (95% CI 1.54–5.86) compared with VDG1 (Table 3). Both visually assessed density and Volpara percent density showed a dose response relationship with increasing density (χ2 trend 42.7, *p* < 0.001 and 13.8, *p* < 0.001, respectively). For Densitas and Quantra, those with percentage density in the highest quintile had ORs of 2.34 (95% CI 1.50–3.68) and 1.32 (95% CI 0.85–2.05), respectively (Table 2, Fig. 2). VAS predicted breast cancer risk significantly better than all other density methods in the subset of women who had density measured by all four methods (Table 2, Additional file 1: Table S2). The matched concordance index for VAS was 0.647 (95% CI 0.607–0.688) demonstrating better discrimination between cases and controls than all other methods (Table 4).

## Discussion

Visual assessment of breast density recorded on a VAS was the strongest predictor of breast cancer risk, both in the contralateral breast of women with screen-detected cancers and in the average of bilateral mammographic views prior to the detection of cancer. It is unlikely that the presence of cancer influenced visual assessment in study 1, since a blinded re-read of images from the contralateral breast by four readers showed no evidence of bias [23] and the ORs were similar to those in study 2. There is strong association between the VAS and breast cancer despite known inter-observer variability [32]; since the average VAS score of two readers was used it is likely that cases falling into the top and bottom quintiles of density do so unequivocally.

Volpara and Densitas percent density had the next strongest associations with cancer in both studies, with categorisation into VDG having the largest odds ratio. Volpara, Quantra and Cumulus did not have as strong an association with breast cancer in study 1 as previously reported [13]. This may be due to differences in the approach used; Eng et al. analysed 414 cases from one hospital and 685 unmatched controls from a screening service based in London, adjusting for age, BMI and reproductive variables in the analysis, whilst we analysed 366 cases using Volpara and Quantra, and 311 using Cumulus, with 3 well-matched controls per case all recruited from the same screening programme. There were also a number of differences between the study populations, with our study population tending to be younger, with more women of white ethnicity and with higher BMI and being less likely to be postmenopausal and to have had children. Density distributions also differed across the two studies, with the current study having lower median (IQR) percent density assessed by Volpara (4.9, 3.5–7.4) and Quantra (11, 8–14), but higher percent density for Cumulus (20.3, 11.6–30.3) [13]. Our version of Volpara was later (1.5.0 vs 1.0) and we applied a Volpara macro for outlier rejection; our version of Quantra was also more recent (2.0 vs 1.3). For Cumulus, the difference might be due to reader experience.

Study 2 examined the relationship between mammographic density in mammograms prior to the detection of cancer, and in matched controls that subsequently remained cancer free. This enables us to evaluate which mammographic density methods are most appropriate for stratifying women attending breast screening. Whilst visual assessment was most strongly associated with cancer, it is unlikely to be used widely for population-based stratified screening; we conclude that Volpara or Densitas percentage density provide a pragmatic solution. However, we hypothesise that methods that measure purely the quantity or relative proportion of dense tissue do not fully capture the mammographic risk in the same way as visual assessment by experts, who can see not only the quantity of dense tissue but the location and pattern. The addition of algorithms that automatically quantify mammographic pattern to automated density software could potentially provide a solution that more closely reproduces visual assessment. Recent research in this area has proved promising [33–36], although there is as yet no consensus as to the best method of encapsulating texture information within risk assessment.

### Strengths and limitations

The strengths of this study include the ability to assess the relationship between several measures of mammographic density and risk of breast cancer. As well as examining the association between mammographic density and breast cancer risk, we were able to establish the temporal relationship in study 2. We also gathered detailed information in relation to a number of covariates (demographic, hormonal, reproductive, lifestyle and family history) via a self-reported questionnaire at entry to PROCAS [20]. Uptake to PROCAS was relatively low (38%), which may have biased the population to those with higher or lower risk, for example, the proportion of women in the PROCAS study who were overweight or obese was significantly lower than in the general population of Greater Manchester [37]. In addition, in study 1, due to the nature of the study design, whereby controls had to have had a subsequent cancer-free mammogram after entry to the PROCAS study, the year of mammogram in controls tended to be earlier than in cases, this may have had an impact on density measures due to changes in mammography technology, and the use of different mammographic machines over time.

## Conclusions

Visual assessment of density, recorded on a VAS and averaged between two independent readers, is a strong predictor of breast cancer risk both in mammograms taken before the detection of cancer and in images of the opposite breast at the time of detection. Percentage density measured by Volpara and Densitas also showed a strong association with breast cancer risk amongst the automated measures evaluated, providing practical automated methods for risk stratification in personalized screening programmes.

## Additional file

Additional file 1: Table S1. Risk of developing breast cancer using continuous measures of different density methods (OR per SD). **Table S2.** *P* values based on likelihood ratio comparing different models for density methods using the subset of those with data on all methods. (DOCX 26 kb)

## Abbreviations

BIRADS: Breast Imaging Reporting And Data System
BMI: Body mass index
CI: Confidence interval
FFDM: Full- field digital mammography
HRT: Hormone replacement therapy
IQR: Interquartile range
ICC: Intraclass correlation coefficient
MLO: Mediolateral oblique
OR: Odds ratio
PROCAS: Predicting Risk of Cancer At Screening Study
VAS: Visual analogue scale
VDG: Volpara Density Grade
