# Supplementary material for: A comparison of five methods of measuring mammographic density: a case-control study
Source: Breast Cancer Res. 2018 Feb 5;20:10. doi: 10.1186/s13058-018-0932-z (PMC5799922; doi:10.1186/s13058-018-0932-z)
Supplement: Additional file 1: Table S1. — Risk of developing breast cancer using continuous measures of different density methods (OR per SD). Table S2. P values based on likelihood ratio comparing different models for density methods using the subset of those with data on all methods. (DOCX 26 kb) [file 13058_2018_932_MOESM1_ESM.docx]

| **Table S1: Risk of developing breast cancer using continuous measures of different density methods (OR per SD)** | | | | | | | | | | | |  |
| --- | --- | --- | --- | --- | --- | --- | --- | --- | --- | --- | --- | --- |
|  |  |  |  |  |  |  | **Subset with data for all methods^a^** | | | | |  |
|  | **Univariate** | |  | **Adjusted^b^** | |  | **Univariate** | |  | **Adjusted^b^** | |  |
|  |  |  |  |  |  |  |  |  |  |  |  |  |
|  | **OR** | **95% CI** |  | **OR** | **95% CI** |  | **OR** | **95% CI** |  | **OR** | **95% CI** |  |
| **Study 1** |  |  |  |  |  |  |  |  |  |  |  |  |
|  | 1.00 | (referent) |  | 1.00 | (referent) |  | 1.00 | (referent) |  | 1.00 | (referent) |  |
| **Square root VAS (%)** | *1.67* | *(1.45-1.94)* |  | *1.66* | *(1.44-1.92)* |  | *1.73* | *(1.49-2.02)* |  | *1.71* | *(1.47-2.00)* |  |
| **Logarithm volpara gland volume (cm^3^)** | *1.31* | *(1.15-1.48)* |  | *1.30* | *(1.14-1.47)* |  | *1.25* | *(1.09-1.44)* |  | *1.25* | *(1.08-1.44)* |  |
| **Logarithm volpara breast density (%)** | *1.38* | *(1.20-1.59)* |  | *1.38* | *(1.20-1.58)* |  | *1.38* | *(1.18-1.61)* |  | *1.38* | *(1.18-1.61)* |  |
| **Sqaure root cumulus dense area (cm^2^)** | *1.21* | *(1.06-1.39)* |  | *1.22* | *(1.06-1.39)* |  | *1.21* | *(1.06-1.40)* |  | *1.22* | *(1.06-1.40)* |  |
| **Square root cumulus percent density (%)** | *1.31* | *(1.13-1.52)* |  | *1.31* | *(1.13-1.53)* |  | *1.33* | *(1.14-1.56)* |  | *1.34* | *(1.14-1.57)* |  |
| **Logarithm quantra gland volume (cm^3^)** | 0.99 | (0.87-1.13) |  | 0.97 | (0.85-1.11) |  | 0.96 | (0.82-1.11) |  | 0.95 | (0.81-1.10) |  |
| **Logarithm quantra breast density (%)** | 1.02 | (0.90-1.16) |  | 1.01 | (0.89-1.15) |  | 1.03 | (0.90-1.19) |  | 1.02 | (0.89-1.18) |  |
| **Logarithm densitas dense area (cm^2^)** | *1.16* | *(1.02-1.31)* |  | *1.14* | *(1.00-1.29)* |  | 1.14 | (0.99-1.32) |  | 1.13 | (0.98-1.31) |  |
| **Logarithm densitas breast density (%)** | *1.36* | *(1.18-1.57)* |  | *1.34* | *(1.16-1.54)* |  | *1.35* | *(1.16-1.57)* |  | *1.33* | *(1.14-1.54)* |  |
|  |  |  |  |  |  |  |  |  |  |  |  |  |
| **Study 2** |  |  |  |  |  |  |  |  |  |  |  |  |
|  | 1.00 | (referent) |  | 1.00 | (referent) |  | 1.00 | (referent) |  | 1.00 | (referent) |  |
| **Square root VAS (%)** | *1.71* | *(1.49-1.98)* |  | *1.73* | *(1.49-2.00)* |  | *1.73* | *(1.49-2.00)* |  | *1.74* | *(1.50-2.02)* |  |
| **Logarithm volpara gland volume (cm^3^)** | *1.43* | *(1.26-1.63)* |  | *1.41* | *(1.24-1.62)* |  | *1.41* | *(1.23-1.61)* |  | *1.39* | *(1.21-1.59)* |  |
| **Logarithm volpara breast density (%)** | *1.43* | *(1.24-1.66)* |  | *1.45* | *(1.24-1.68)* |  | *1.41* | *(1.22-1.64)* |  | *1.43* | *(1.22-1.66)* |  |
| **Logarithm quantra gland volume (cm^3^)** | *1.32* | *(1.04-1.68)* |  | 1.27 | (0.99-1.62) |  | *1.16* | *(1.01-1.34)* |  | 1.14 | (0.98-1.31) |  |
| **Logarithm quantra breast density (%)** | *1.17* | *(1.02-1.34)* |  | *1.17* | *(1.02-1.34)* |  | *1.16* | *(1.01-1.33)* |  | *1.16* | *(1.01-1.34)* |  |
| **Logarithm densitas dense area (cm^2^)** | *1.32* | *(1.16-1.51)* |  | *1.30* | *(1.14-1.48)* |  | *1.32* | *(1.16-1.51)* |  | *1.30* | *(1.14-1.48)* |  |
| **Logarithm densitas breast density (%)** | *1.37* | *(1.18-1.58)* |  | *1.35* | *(1.16-1.57)* |  | *1.36* | *(1.18-1.58)* |  | *1.35* | *(1.16-1.56)* |  |

**^a^Study 1: 239 cases with 3 controls, 62 with 2 controls and 2 with 1 control; Study 2: 296 cases with 3 controls, 31 with 2 controls and 2 with 1 control**

**^b^Adjusted for Tyrer-Cuzick score; Study 2 also adjusted for parity**

**OR – odds ratio; SD – standard deviation; CI – confidence interval; VAS – Visual Analogue Scale. Italics indicate statistically significant results (p<0.05)**

| **Table S2: P-values based on likelihood ratio comparing different models for density methods using the subset of those with data on all methods*** | | | | | | | | | | | |
| --- | --- | --- | --- | --- | --- | --- | --- | --- | --- | --- | --- |
|  | **VAS** | **Volpara** | | **Quantra** | | **Densitas** | | **Cumulus** | |  |  |
|  | **%** | **%** | **GV** | **%** | **GV** | **%** | **DA** | **%** | **DA** |  |  |
| **Study 1** |  |  |  |  |  |  |  |  |  |  |  |
|  |  |  |  |  |  |  |  |  |  |  |  |
| VAS (%) | - | *<0.001* | *<0.001* | *<0.001* | *<0.001* | *<0.001* | *<0.001* | *<0.001* | *<0.001* |  |  |
| Volpara breast density (%) | | - | 0.136 | *0.006* | *0.008* | 0.345 | *0.018* | 0.240 | 0.055 |  |  |
| Volpara gland volume (cm^3^) | |  | - | *0.013* | 0.067 | 0.791 | *0.019* | 0.695 | 0.289 |  |  |
| Quantra breast density (%) | |  |  | - | 0.517 | 0.994 | 0.866 | 0.994 | 0.965 |  |  |
| Quantra gland volume (cm^3^) | |  |  |  | - | 0.990 | 0.770 | 0.989 | 0.920 |  |  |
| Densitas breast density (%) | |  |  |  |  | - | *0.013* | 0.358 | 0.085 |  |  |
| Densitas dense area (cm^2^) | |  |  |  |  |  | - | 0.957 | 0.859 |  |  |
| Cumulus breast density (%) | |  |  |  |  |  |  | - | *0.031* |  |  |
| Cumulus dense area (cm^2^) | |  |  |  |  |  |  |  | - |  |  |
|  |  |  |  |  |  |  |  |  |  |  |  |
| **Study 2** |  |  |  |  |  |  |  |  |  |  |  |
|  |  |  |  |  |  |  |  |  |  |  |  |
| VAS (%) | - | *0.001* | *0.009* | *<0.001* | *<0.001* | *<0.001* | *0.001* | - | - |  |  |
| Volpara breast density (%) | | - | 0.726 | *<0.001* | *0.015* | 0.270 | 0.372 | - | - |  |  |
| Volpara gland volume (cm^3^) | |  | - | *<0.001* | *<0.001* | 0.170 | 0.067 | - | - |  |  |
| Quantra breast density (%) | |  |  | - | 0.471 | 0.995 | 0.982 | - | - |  |  |
| Quantra gland volume (cm^3^) | |  |  |  | - | 0.972 | 0.998 | - | - |  |  |
| Densitas breast density (%) | |  |  |  |  | - | 0.504 | - | - |  |  |
| Densitas dense area (cm^2^) | |  |  |  |  |  | - | - | - |  |  |
| * Study 1: 239 with 3 controls, 62 with 2 controls, 2 with 1 control; Study 2: 296 with 3 controls, 31 with 2 controls, 2 with 1 control  VAS – Visual Analogue Scale; GV – gland volume; DA – dense area. Italics indicate statistically significant results (p<0.05) | | | | | | | | | | | |
